# Inhibition of Key Digestive Enzymes Related to Diabetes and Hyperlipidemia and Protection of Liver-Kidney Functions by Trigonelline in Diabetic Rats

**DOI:** 10.3797/scipharm.1211-14

**Published:** 2012-12-20

**Authors:** Khaled Hamden, Kais Mnafgui, Zahra Amri, Ahmed Aloulou, Abdelfattah Elfeki

**Affiliations:** 1 Biotechnology High School of Sfax (ISBS),University of Sfax, Soukra Km 45; PO Box 261, Sfax 3038, Tunisia.; 2 Laboratory of Animal Ecophysiology, University of Sfax, Faculty of Sciences of Sfax, PO Box 95, Sfax 3052, Tunisia.; 3 Laboratory of Biochemistry and Enzymatic Engineering of Lipases, National School of Engineers of Sfax, University of Sfax, Sfax 3038, Tunisia.

**Keywords:** Trigonelline, Digestive enzymes, Pancreas, Lipase, Diabetes

## Abstract

Diabetes is a serious health problem and a source of risk for numerous severe complications such as obesity and hypertension. Treatment of diabetes and its related diseases can be achieved by inhibiting key digestive enzymes related to starch and lipid digestion. The findings revealed that the administration of trigonelline to surviving diabetic rats helped to protect the pancreas β-cells from death and damage. Additionally, the supplement of trigonelline to surviving diabetic rats significantly decreased intestinal α-amylase and maltase by 36 and 52%, respectively, which led to a significant decrease in the blood glucose rate by 46%. Moreover, the administration of trigonelline to surviving diabetic rats potentially inhibited key enzymes of lipid metabolism and absorption such as lipase activity in the small intestine by 56%, which led to a notable decrease in serum triglyceride (TG) and total cholesterol (TC) rates and an increase in the HDL cholesterol level. This treatment also improved glucose, maltase, starch, and lipid oral tolerance. Trigonelline was also observed to protect the liver-kidney functions efficiently, which was evidenced by the significant decrease in the serum aspartate transaminase (AST), alanine transaminase (ALT), gamma-glutamyl transpeptidase (GGT), and lactate dehydrogenase (LDH) activities and creatinine, albumin, and urea rates. The histological analysis of the pancreas, liver, and kidney tissues further established the positive effect of trigonelline. Overall, the findings presented in this study demonstrate that the administration of trigonelline to diabetic rats can make it a potentially strong candidate for industrial application as a pharmacological agent for the treatment of hyperglycemia, hyperlipidemia, and liver-kidney dysfunctions.

## Introduction

Diabetes mellitus is a major and growing public health problem throughout the world, with an estimated worldwide prevalence in 2008 of more than of 347 million people and is a heterogeneous disorder with varying prevalence among different ethnic groups and it is reported to constitute the 16th leading cause of global mortality [1]. This disease is particularly characterized by the excessive accumulation of free glucose in blood, which is likely to increase the risk for developing various metabolic disorders, including hyperlipidemia, liver-kidney dysfunctions, and hypertension [2]. One of the therapeutic approaches used to decrease drug-treated diabetes and its complications is to retard the absorption of glucose and lipid through the inhibition of carbohydrate-hydrolyzing enzymes, i.e., α-amylase, maltase, and lipase in the intestine [3–5]. There are reports on the use of fenugreek seed extracts in diabetes mellitus [6, 7]. The seeds of this plant have been used as a traditional remedy for conditions including gastrointestinal disorders, gout, wound healing, inflammation, hyperlipidemia, and diabetes. The biological and pharmacological actions of fenugreek are attributed to the variety of its constituents, namely: steroids as diosgenin, alkaloids as trigonelline [6, 7], and amino acids as 4-Hydroxyisoleucine [8]. In fact, it is reported that the purification alkaloid (GII) from fenugreek and other compounds from fenugreek seeds were demonstrated to be useful in subdiabetic, moderately diabetic, and severely diabetic rabbits [9, 10]. Also, clinical studies have been shown to stimulate glucose-dependent insulin release from isolated rat and human islets [11, 12]. The present results confirm earlier published data by Moorthy *et al*. [9, 10], which reported that the administration of isolated trigonelline from fenugreek to diabetic rats corrects various metabolic enzymes such as enzymes of glycolysis, gluconeogenesis, glycogen metabolism, and the polyol pathway. The new aspect in our present study is the evaluation of the effect of trigonelline on key enzymes related to diabetes and obesity as α-amylase, maltase, and lipase in alloxan-induced diabetic rats.

## Results and Discussion

### Effect of trigonelline on α-amylase and maltase activities and glucose, maltose, and the starch oral tolerance test on diabetic rats

Diabetes mellitus, with its attendant complications and related diseases, constitutes one of the most serious, costly, and fast-growing health problems in the twenty-first century. One of the therapeutic approaches proposed so far for the decrease of drug-treated diabetes is based on the retardation of glucose absorption through the inhibition of carbohydrate-hydrolyzing enzymes, *i.e.*; α-amylase and maltase in the intestine [6].

The results revealed that diabetes induced a considerable increase in the α-amylase, and maltase activities in the mucosal small intestine by 204 and 290% respectively, which led to an increase of the glucose rate by 236% in the serum of diabetic rats. However, in trigonelline -treated diabetic rats, the activities of those enzymes underwent considerable improvements. In fact, the administration of trigonelline to surviving diabetic rats significantly reduced the activities of all of those enzymes in the intestine of surviving diabetic rats. The inhibitory effect on intestinal α-amylase and maltase activities significantly decreased the glucose concentration in the serum (Figure. 2). In fact, the inhibitory effects of α-amylase and maltase seem to have limited the process of carbohydrate hydrolysis and absorption in the intestine, which led to a decrease in serum glucose levels [13, 14]. These results were performed by the oral glucose (3A), maltose (3B), and starch (3C) tolerance tests in conscious fasted rats after trigonelline administration. This study reported that the administration of trigonelline to surviving diabetic rats ameliorated the oral glucose, starch, and maltose tolerance test, establishing the inhibitory effect on the key enzymes related to hyperglycemia. These results clearly showed that the acute oral administration of trigonelline to surviving diabetic rats significantly reduced the peak glucose concentration 60 min after glucose, starch, and maltose administration as compared to untreated diabetic rats (Figure 3), which stands in agreement with findings recently reported in the literature [7]. In fact, a recent study by Puri et *al*[7] reported that the administration of trigonelline purified from fenugreek improves blood glucose utilization and reduces fasting blood sugar and this increased the absolute body, kidney, and liver weights.

### Effect of trigonelline on pancreas architecture and body, liver and kidneys weights diabetic rats

**Fig. 1:**
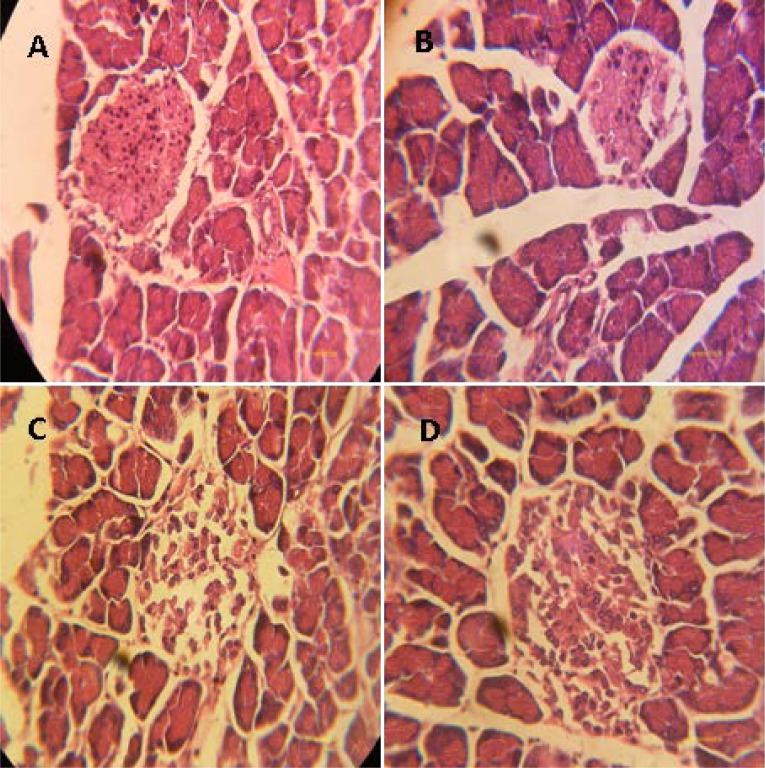
Effect of trigonelline on the histological changes of rats’ pancreas by HE staining. (A): Normal control rats showed normal β-cells; (B): Severe injury in β-cells in the pancreas of male rats given alloxan for 1 month; (C): In diabetic rats treated with Acar: any ameliorative actions were observed compared to diabetic rats after 4 weeks of alloxan; (D): Pancreatic β-cells showing protective effect in diabetic rats treated with trigonelline.

**Fig. 2:**
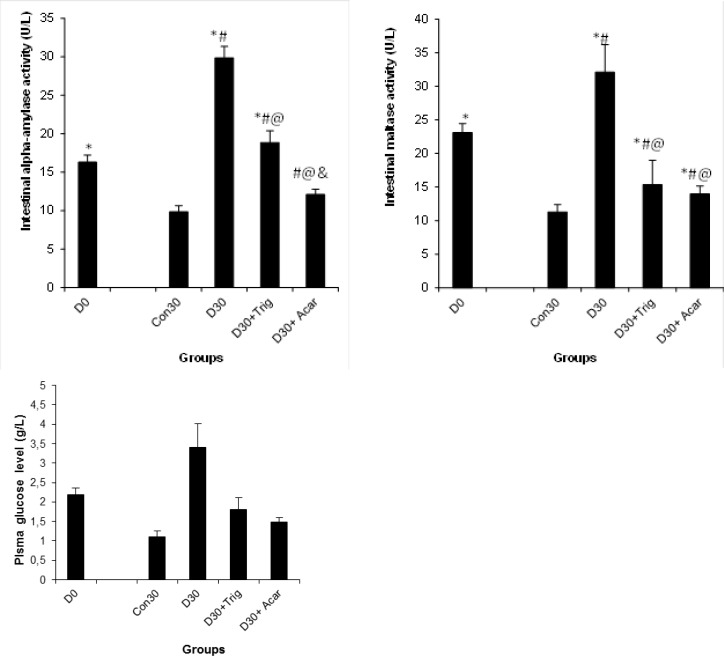
Effect of the trigonelline on small intestine α-amylase and maltase activities and serum glucose level of the control and experimental groups of rats. In diabetic rats, a considerable increase in intestinal α-amylase and maltase activities; which leads to an increase in the plasma glucose rate. However, the administration of trigonelline potentially inhibited amylase and maltase activities and this led to a decrease in plasma glucose rate. ^*^ P < 0.05 significant differences compared to controls. ^#^ P < 0.05 significant differences compared to D0. ^@^ P < 0.05 significant differences compared to D30. ^&^ P < 0.05 significant differences compared to D30+Trig.

**Fig. 3:**
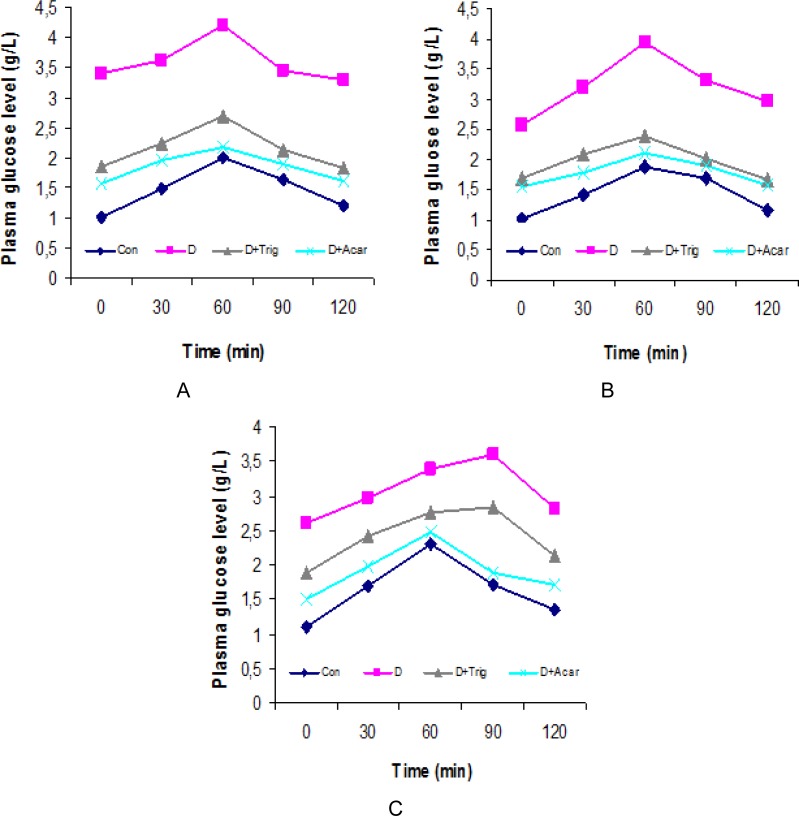
Oral glucose, starch, and maltose tolerance tests on the control and experimental groups of rats. Results of this study revealed that administration of trigonelline significantly reduced the peak glucose the same as ameliorated insulin sensibility in surviving diabetic rats. ^*^ P < 0.05 significant differences compared to controls. ^#^ P < 0.05 significant differences compared to D0. ^@^ P < 0.05 significant differences compared to D30. ^&^ P < 0.05 significant differences compared to D30+Trig.

Figure 1 presents the histopathological examination of the pancreas. In the control rat, the pancreas showed normal islets (Figure 1A). In alloxan-treated rats, pancreases showed severe β-cells atrophy (Figure 1B). In trigonelline-treated diabetic rats (Figure 1D), a partial protective action of β-cells was observed and only initial stages of atrophy of β-cells were observed. In fact, it wase reported that the administration of trigonelline to surviving diabetic rats restored the structure of pancreas β-cells resulting in the increase of insulin secretion which decreased glucose levels in serum [15]. The protection of pancreas architecture led to an increase in insulin rate, and consequently, the regulation of glucose metabolism and an increase in body, hepatic, and renal weights (Table 1). These results, in agreement with findings recently reported by Puri et *al*[6]), have reported that the administration of fenugreek trigonelline to diabetic rats modulates key enzymes of metabolism such as hexokinase, glucokinase, pyruvate kinase, malic enzyme, glucose-6-phosphate dehydrogenase, glucose-6-phosphatase, sorbitol dehydrogenase, and aldose reductase, and also the glucogen rate in muscle and the liver.

### Effect of trigonelline on lipase activity in the serum and intestine; oral lipid tolerance test in diabetic rats

The findings of the present study also demonstrated that diabetes increased lipase activity in the intestine and that the increased lipid absorption from the intestine consequently amplified the hypercholesterolemia and hyperlipidemia in the serum. The administration of trigonelline to surviving diabetic rats also exerted *in vivo* inhibitory effects on the key enzymes of lipid digestion and absorption such as lipase in the small intestine, which contributes therapeutic action against hyperlipidemia, obesity, and heart diseases [16]. In fact, this enzyme is secreted from the pancreas, transported to the small intestine, and hydrolyzes non-absorbable triglycerides into simple glycerol and fatty acids absorbable by the small intestine. Therefore, intestinal lipase inhibitors are considered to be a valuable therapeutic reagent for treating diet-induced obesity in humans.

**Fig. 4:**
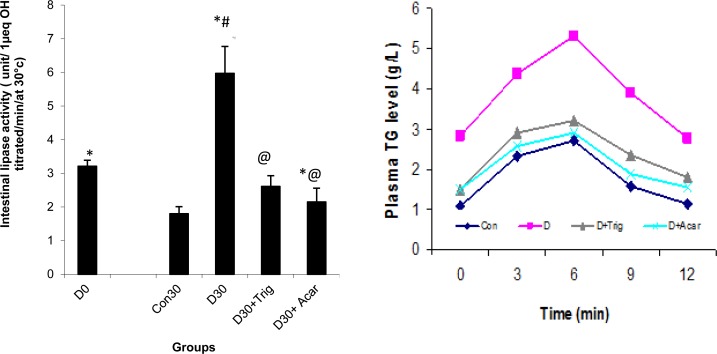
Activities of lipase in the small intestine and the oral oil tolerance test of the control and experimental groups of rats. In diabetic rats, there is a significant increase in lipase activity which leads to disorders in the lipid profile. However, the administration of trigonelline regulates lipase activity and maintains the lipid profile. ^*^ P < 0.05 significant differences compared to controls. ^#^ P < 0.05 significant differences compared to D0. ^@^ P < 0.05 significant differences compared to D30. ^&^ P < 0.05 significant differences compared to D30+Trig.

This study indicated in Figure 4 that the administration of trigonelline to surviving diabetic rats, on the other hand, clearly reverted the activity of intestinal lipase nearly back to that of the non-diabetic rats. The inhibitory action of lipase in the intestine decreased the hydrolysis of dietary triglycerides into monoglycerides and free fatty acids as it lowered the TC, LDL-C, and TG and it increased of HDL-C levels in serum (Table 1).

This study was performed by an oral lipid tolerance test and was performed in all rats at the end of this treatment. Rats were fasted for 12 h before the test and serum samples were taken from the tail vein for the determination of TG levels. Then, an oral oil challenge (2 g/kg body weight) was given by gavage and serum samples were taken at 3, 6, 9, and 12 hours after oil administration. The serum TG level was increased after 3 and 6 h oral administration of the trigonelline, this being followed by a decrease 6 h after the administration, with its effect being the strongest at 9 h after the oil gavage. These results are in agreement with previous studies reported that fenugreek trigonelline decreased the elevated lipids TC, TG, LDL-C, and increased the decreased HDL-C [6, 16].

### Effect of trigonelline on liver-kidney functions on diabetic rats

**Fig. 5:**
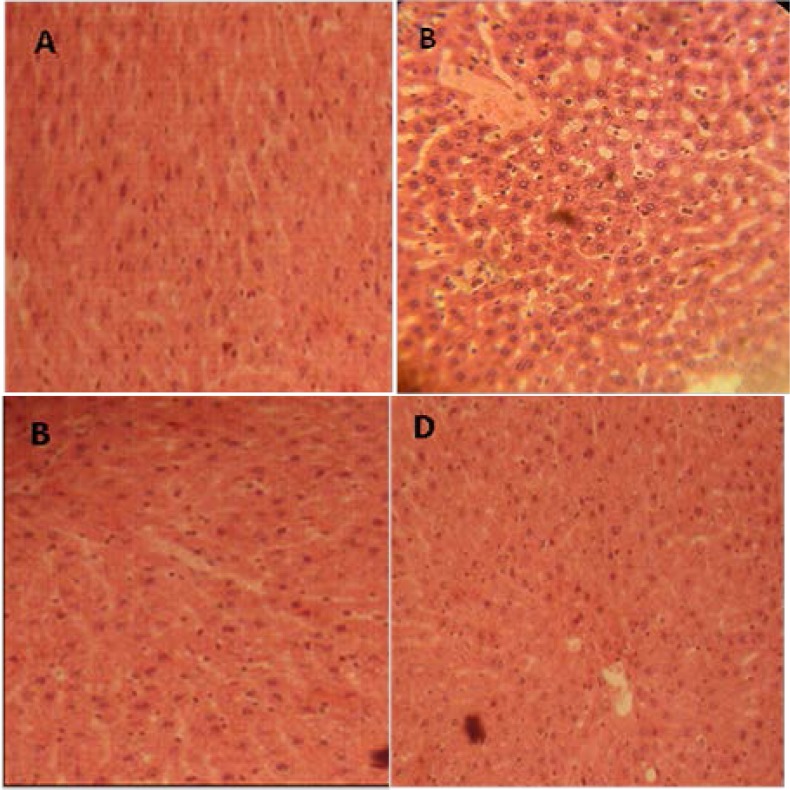
Histopathological studies of the liver in the control and experimental groups of rats. Section of the liver from A) control rats; B) diabetic rats at day 30 showing fatty cysts apparition in liver tissues; C) In diabetic rats treated with Acar: a few ameliorative actions were observed compared to diabetic rats after four weeks of alloxan D): diabetic rats treated respectively with trigonelline, protective action was shown.

As far as the liver of diabetic rats is concerned, the present study showed an increase in terms of the AST, ALT, LDH, and GGT activities by 88, 83, 63, and 44%, respectively, in serum (Table). Interestingly, the administration of trigonelline to surviving diabetic rats seems to have reverted back this increase and ameliorated all indices related to liver dysfunction induced by diabetes. Findings from further histological analysis were found to confirm the positive effects of trigonelline as shown in Figure 5B, where fatty cysts, indicated by the arrow, appeared in the hepatic tissues of diabetic rats as compared to normal rats. However, the administration of trigonelline to surviving diabetic rats protected liver tissues (Figure 5D).

**Fig. 6:**
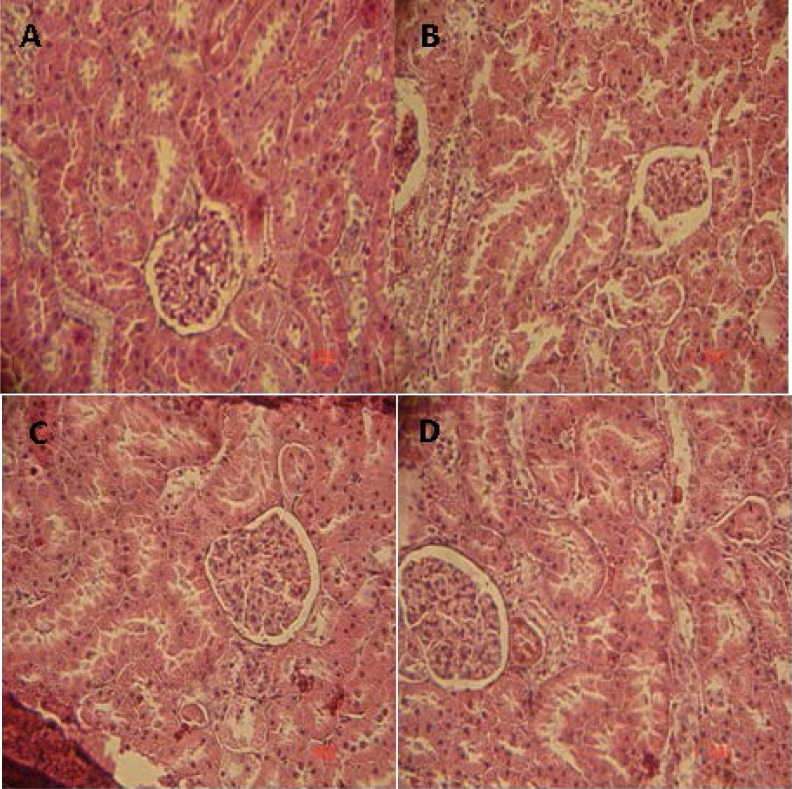
Histopathological studies of kidney in the control and experimental groups of rats. Section of the kidney from A) control rats; B) diabetic rats at day 30 showing histopathological changes (e.g. capsular space shrinkage and glomerular hypertrophy); C, D): diabetic rats treated respectively with Acar and trigonelline, protective action was shown.

Moreover, the table evidenced that the administration of the trigonelline to surviving diabetic rats seems to have reverted back this increase of serum creatinine and urea, with a significant decrease in albumin as compared to untreated diabetic rats. The positive effect was confirmed by histological findings. In fact, in Figure 6B, diabetic rats at day 30 showed histopathological changes (e.g. capsular space shrinkage and glomerular hypertrophy) as compared to control rats (Figure 6A); however, after Acar or trigonelline administration to surviving diabetic rats (Figure 6C, D) a potential protective action was shown. These results agree with few studies showing that fenugreek trigonelline administration corrected most of the abnormalities seen in the liver and kidneys of the untreated diabetic animals as fatty infiltration and other cellular changes [6, 17].

In conclusion, the present study has demonstrated that trigonelline significantly improved glucose and lipid homeostasis in diabetes by delaying carbohydrate and lipid digestion and absorption, and enhancing or mimicking insulin action. Therefore, trigonelline represents a potentially useful dietary adjunct for the treatment of diabetes, obesity, and a potential source for the discovery of new orally active antidiabetic agents)

## Materials and methods

### Animals and treatments

The assays of the present study were conducted on adult male *Wistar* rats, weighing 153 ± 12 g, which were obtained from the local Central Pharmacy, Tunisia. All rats were kept in an environmentally controlled breeding room, temperature: 20 ± 2°C, humidity: 60 ± 5%, 12-h dark/light cycle) where they had standard diets and free access to tap water. The experimental protocols were conducted in accordance with the Guide for the Care and Use of Laboratory Animals issued by the University of Sfax, Tunisia, and approved by the Tunisia Committee of Animal Ethics.

Diabetes was induced in rats by a single intraperitoneal injection of freshly prepared alloxan solution in normal saline at a dose of 150 mg/kg body weight (6). One week later, the blood glucose concentration was measured, and the value was two-fold higher than that in the control rats. The serum glucose level of the rats was measured by the glucose-oxidase reaction using commercially available kits from Biomagreb Tunis, Tunisia. On the day the experiments started, and before treatment, the rats were divided into eight groups of eight animals each as follows:

Group 1: diabetic rats named at day 0 of this experimentation (D_0_).
Group 2: diabetic control rats (D_30_).
Group 3: normal rats named (Con).
Group 4: diabetic rats treated with the formulation Trig by the gastric gavage route 50 mg/kg of body weight/day during 30 days) and termed (D_30_+ trigonelline).
Group 5: diabetic rats treated with acarbose by the gastric gavage route 10 mg/kg of body weight/day during 30 days) and termed (D_30_+Acar).

One month later, the rats were weighed and sacrificed by decapitation, and their trunk blood was collected. The serum was prepared by centrifugation 1,500 ×g, 15 min, 4°C).

### Biochemical analysis

Trigonelline, alloxan, maltose, starch, and glucose were purchased from Sigma-Aldrich St. Louis, MO, USA), the GOD, HDL, TC, TG, AST, ALT, LDH, GGT, LDH, creatinine, albumin, and urea were from Biomaghreb Analyticals Tunis, Tunisia). All other chemicals used were of analytical grade. The mucosal small intestine of each rat was excised and the lumen was flushed out several times with 0.9% NaCl. The mucosal washing and the scraped mucosa were pooled, homogenized, and centrifuged 5,000 × g, 15 min). The supernatant was frozen and stored at −80°C for further use in subsequent enzymatic assays. The activities of α-amylase and maltase were obtained by measuring the amount of glucose released from various substrates [18]. Lipase activity was measured by the assay described previously [19]. For the oral sugar and lipid tolerance test, the carbohydrates loaded were as follows: glucose 2 g/kg), maltose 2 g/kg), starch 1 g/kg), and oil 2 g/kg). These carbohydrates were orally administered via a gastric gavage route. Blood samples were collected from the tail vein at 0, 05, 1, and 2 h after the carbohydrate and oil administration. For histochemical procedures, tissue specimens of the pancreas, liver, and kidney were obtained and fixed with 10% buffered formalin, and subsequently embedded in paraffin. After that, the paraffin-embedded samples were cut in sections, thickness 5 μm and then stained with hematoxylin-eosin. The samples were then examined using an Olympus CX41 light microscope.

### Statistical analysis

The values are means ± SD. Determinations were performed from eight animals per group and differences were examined by a one-way analysis of variance (ANOVA) followed by the Fisher test (Stat View). The significance was accepted at p < 0.05.

## Figures and Tables

**Tab. 1.** Effect of trigonelline on absolute and relative body, liver, and kidneys weights, liver indices toxicity (AST, ALT, LDH, and GGT activities), kidney indices dysfunctions (creatinine, urea, and albumin rates) and lipid profile (T-C, TG, and LDH-C levels) in plasma of diabetic rats.

	**D_0_**	**Con_30_**	**D_30_**	**D_30_+Trig**	**D_30_+Acar**
Body weight	130 ± 15^*^	215 ± 12	166 ± 13^*#^	212 ± 11^#@^	203 ± 14^#@^
Liver weight	7.3 ± 1.4^*^	11.8 ± 1.2	5.81 ± 0.9^*^	9.5 ± 0.9^*#@^	8.9 ± 0.7^*#@^
Kidney weight	0.91 ± 0.06^*^	1.31 ± 0.09	0.71 ± 0.04^*^	1.05 ± 0.07^*@^	0.98 ± 0.05^*&^
Relative liver weight (%)	5.6 ± 0.81	5.48 ± 0.21	3.5 ± 0.31^*#^	4.48 ± 0.26^*#@^	4.38 ± 0.18^*#@^
Relative kidney weight (%)	0.7 ± 0.04^*^	0.6 ± 0.03	0.42 ± 0.06^#^	0.49 ± 0.04^*#^	0.48 ± 0.023^*#^
AST (U/L)	145 ± 112^*^	109.6 ± 13	179 ± 11^*#^	129.8 ± 4^*#@^	139 ± 8^*@&^
ALT (U/L)	60.1 ± 10^*^	47.8 ± 2.8	88.1 ± 14^*^	55.3 ± 7.3^#@^	59.3 ± 7.3^*@^
LDH (U/L)	1139 ± 97.5^*^	876 ± 31	1435 ± 221^*#^	942 ± 35^*#@^	953 ± 75^*#@^
GGT (U/L)	7.79 ± 1.69	6.76 ± 0.78	9.76 ± 1.91^*^	7.21 ± 1.33^@^	7.96 ± 1.43^*@^
Creatinine (mg/L)	23.9 ± 2.8^*^	20.6 ± 1.3	34.1 ± 2.6^*@^	25.8 ± 1.1^*#@^	28.8 ± 1.8^*#@&^
Urea (g/L)	1.29 ± 0.21^*^	0.84 ± 0.03	1.61 ± 0.26^*#^	1.03 ± 0.09^*#@^	1.13 ± 0.1^*#@&^
Albumin (g/L)	34.1 ± 4.1^*^	24.1 ± 2.1	44.3 ± 2.6^*#^	29.8 ± 2.1^*@^	34.8 ± 3.1^*#@&^
T-Ch (g/L)	1.65 ± 0.16^*^	1.23 ± 0.12	2.30 ± 0.12^*#^	1.41 ± 0.11^#@^	1.59 ± 0.13^@^
HDL-Ch (g/L)	0.59 ± 0.08^*^	0.88 ± 0.06	0.39 ± 0.06^*#^	0.73 ± 0.05^#@^	0.69 ± 0.04^*@^
LDL-Ch (g/L)	0.97 ± 0.19^*^	0.55 ± 0.15	1.81 ± 0.12^*#^	0.83 ± 0.09^*@^	0.79 ± 0.12^*@^
TG (g/L)	1.31 ± 0.31^*^	0.91 ± 0.17	1.59 ± 0.26^*#^	1.04 ± 0.08^#@^	0.98 ± 0.13^#@^

* P < 0.05 significant differences compared to controls.

# P < 0.05 significant differences compared to D0.

@ P < 0.05 significant differences compared to D30.

& P < 0.05 significant differences compared to D30+Trig.
