# Corrigendum to “Inhibition of Key Digestive Enzymes Related to Diabetes and Hyperlipidemia and Protection of Liver-Kidney Functions by Trigonelline in Diabetic Rats” [Sci Pharm. 2013; 81: 233–246]

**DOI:** 10.3797/scipharm.1211-14corr

**Published:** 2014-03-13

**Authors:** Khaled Hamden, Kais Mnafgui, Zahra Amri, Ahmed Aloulou, Abdelfattah Elfeki

**Affiliations:** 1 Biotechnology High School of Sfax (ISBS), University of Sfax, Soukra Km 45; PO Box 261, Sfax 3038, Tunisia.; 2 Laboratory of Animal Ecophysiology, University of Sfax, Faculty of Sciences of Sfax, PO Box 95, Sfax 3052, Tunisia.; 3 Laboratory of Biochemistry and Enzymatic Engineering of Lipases, National School of Engineers of Sfax, University of Sfax, Sfax 3038, Tunisia.

**Keywords:** Corrigendum, Sci Pharm. 2013, 81: 233–246

## Abstract

This is a corrigendum to the article ‘Inhibition of Key Digestive Enzymes Related to Diabetes and Hyperlipidemia and Protection of Liver-Kidney Functions by Trigonelline in Diabetic Rats’ [Sci Pharm. 2013; 81: 233–246]. Figure 6 is replaced.

Unfortunately, Figure 6 incorrectly appeared in the published article due to a numbering problem of the blades used for the pictures [1]. Hence, this wrong figure should be replaced with the correct one given below. The authors are very sorry for this error and for any inconvenience this caused.

**Fig. 6:**
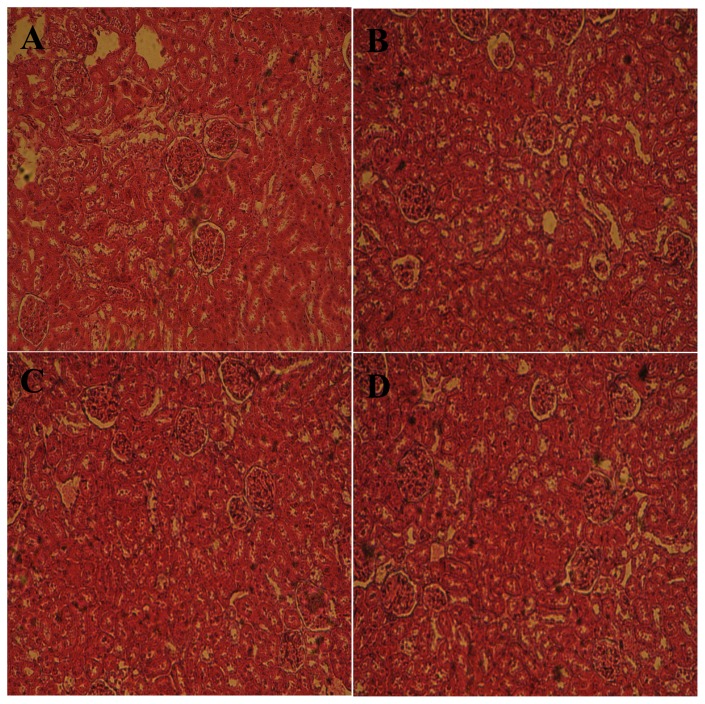
Histopathological studies of the kidney in the control and experimental groups of rats. Section of the kidney from A) control rats; B) diabetic rats at day 30 showing histopathological changes (e.g. capsular space shrinkage and glomerular hypertrophy); C, D): diabetic rats treated respectively with Acar and trigonelline, the protective action was shown.
